# Case Report: Methotrexate and hydroxychloroquine in combination for the treatment of NOD2-mutation-associated Blau syndrome

**DOI:** 10.3389/fimmu.2023.1279329

**Published:** 2023-10-05

**Authors:** Mary Ellen Jensen, Katelin Harrell, Jeffrey D. McBride

**Affiliations:** ^1^ College of Medicine, University of Oklahoma Health Sciences Center, Oklahoma City, OK, United States; ^2^ Department of Dermatology, University of Oklahoma Health Sciences Center, Oklahoma City, OK, United States; ^3^ Department of Pathology, University of Oklahoma Health Sciences Center, Oklahoma City, OK, United States

**Keywords:** Blau syndrome, early-onset sarcoidosis, hydroxychloroquine, methotrexate, arthritis

## Abstract

Mutations in nucleotide binding oligomerization domain containing 2 receptor (NOD2) are associated with Blau syndrome (also known as early-onset sarcoidosis)—a rare autosomal dominant, chronic granulomatous disease that typically presents before 5 years of age. Blau syndrome is characterized by the clinical triad of arthritis, granulomatous dermatitis, and recurrent uveitis. Here, we report a case of NOD2-mutation-associated early-onset sarcoidosis in which a combination of methotrexate and hydroxychloroquine was used to achieve improvement in arthritis, granulomatous dermatitis, and uveitis. A 13-month-old boy presented with a sudden-onset cutaneous eruption affecting the face, trunk, and extremities that initially mimicked papular atopic dermatitis but progressively worsened despite topical steroid therapy. The patient had no other known medical comorbidities or abnormalities except for heterochromia of the right eye. However, prior to presentation to dermatology, the patient began experiencing frequent falls, conjunctival injection, and apparent eye and joint pain. Skin biopsy from the right shoulder demonstrated rounded aggregates of epithelioid histiocytes and multinucleated giant cells without a significant lymphocytic component (“naked granulomas”), consistent with sarcoidal granulomatous dermatitis. Given the concern for Blau syndrome, the patient was sent for evaluation by ophthalmology and was found to have bilateral subconjunctival nodules. Our patient underwent genetic testing and was found to have a mutation in codon 1000 C > T (protein R334W) in the NOD2 gene. The patient responded to oral prednisolone 2 mg/kg/day for 8 weeks, but quickly relapsed, requiring a second 8-week course with taper upon starting methotrexate 7.5 mg subcutaneously weekly with 1 mg folic acid orally daily. After 8 weeks on methotrexate, due to persistent arthritis, conjunctival injection, and pruritus, and in consultation with rheumatology, the patient was started on hydroxychloroquine 75 mg orally daily along with continuation of 7.5 mg methotrexate subcutaneously weekly for 8 weeks, achieving significant reduction in arthritis, pruritus, and uveitis. After 8 weeks of this combination therapy, due to concerns of long-term macular toxicity, hydroxychloroquine was discontinued in favor of continuing methotrexate alone. The patient has remained free of significant side effects and stable with good disease control on 7.5 mg methotrexate weekly injected subcutaneously.

## Introduction

Blau syndrome is a rare autosomal dominant chronic granulomatous disease that typically presents before 5 years of age (hence the alternate name of “early-onset sarcoidosis”). The term “early-onset sarcoidosis” has been consolidated under Blau syndrome in Online Mendelian Inheritance in Man (OMIM) and will thus be referred to as Blau syndrome in this report. Patients with Blau syndrome present with scaly erythematous or flesh-colored fine papules or coalescent plaques. Boggy polyarthritis with exuberant tenosynovitis develops between 2 and 4 years of age (1, 2). It is also seen that patients will have recurrent anterior uveitis along with eye pain, photophobia, and blurred vision (2, 3). Uveitis develops in 60%–80% of patients at approximately 48 months of age (2). Early diagnosis and treatment are critical to preventing progression of joint and eye symptoms. Serious complications including granulomatous uveitis lead to blindness and joint contracture (4, 5).

Blau syndrome is caused by mutations related to NOD2 gene on chromosome 16q12. While the direct correlation to gene mutation and disease manifestation is not fully understood, it is suggested that mutations in NOD2 result in dysfunction of protein oligomerization and subsequent interaction with downstream adaptors (5–8).

NOD2 is a pattern recognition receptor that recognizes bacterial peptidoglycan intracellularly and plays a critical role in host defense and inflammation (6). Specifically, muramyl dipeptide (MDP) is required to activate NOD2 leading to binding of receptor-interacting protein kinase 2 (RIPK2), inducing RIPK2 activation (8). RIPK2 induces a cascade of downstream effects ultimately leading to the activation of NF-kB and mitogen-associated protein kinase (MAPK) signaling pathways (6).

## Case description

**Figure 1:**
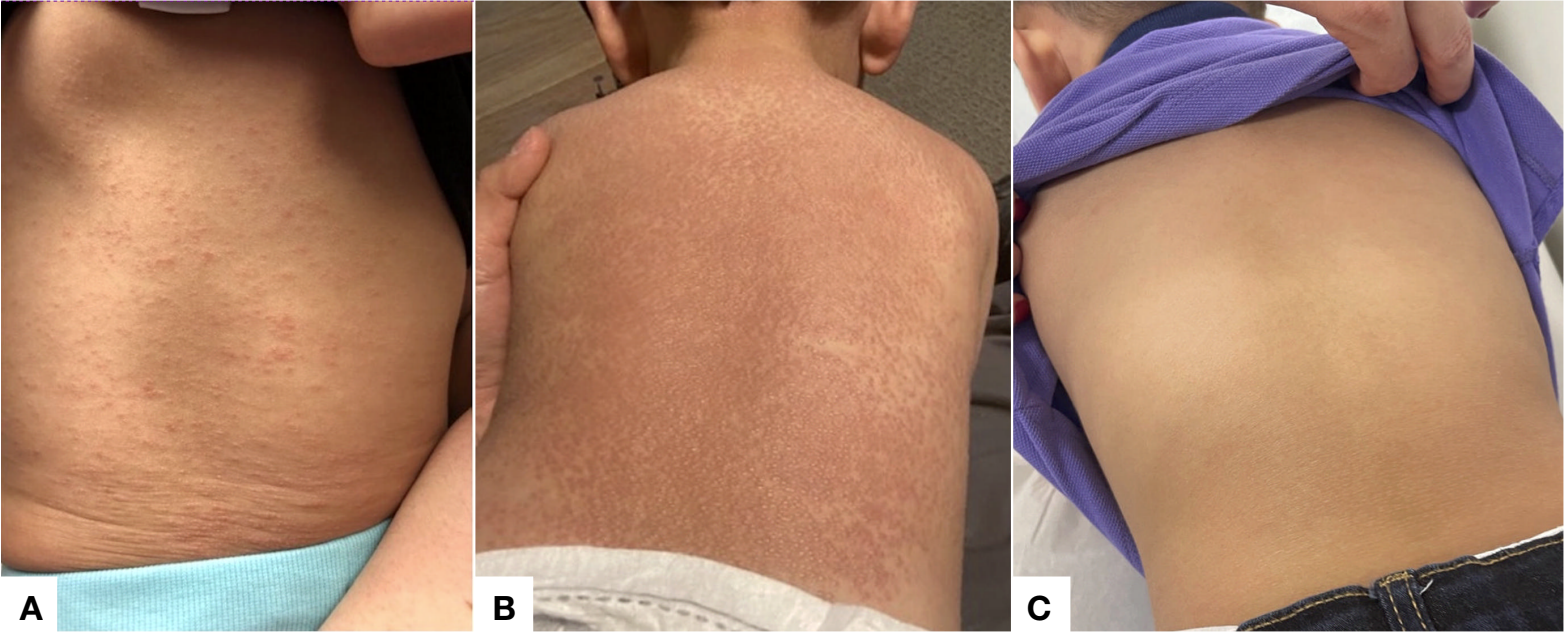
Clinical presentation of cutaneous eruption in Blau syndrome. **(A)** Initial presentation of diffuse scaly erythematous and skin-colored, fine papules located on the patient’s back. **(B)** Two months after initial presentation, the patient had worsening cutaneous papules coalescing into plaques on the patient’s back. **(C)** Follow-up visit approximately 1 year after initial presentation, showing significant improvement on methotrexate and hydroxychloroquine combination therapy for a total of 8 weeks.

**Figure 2:**
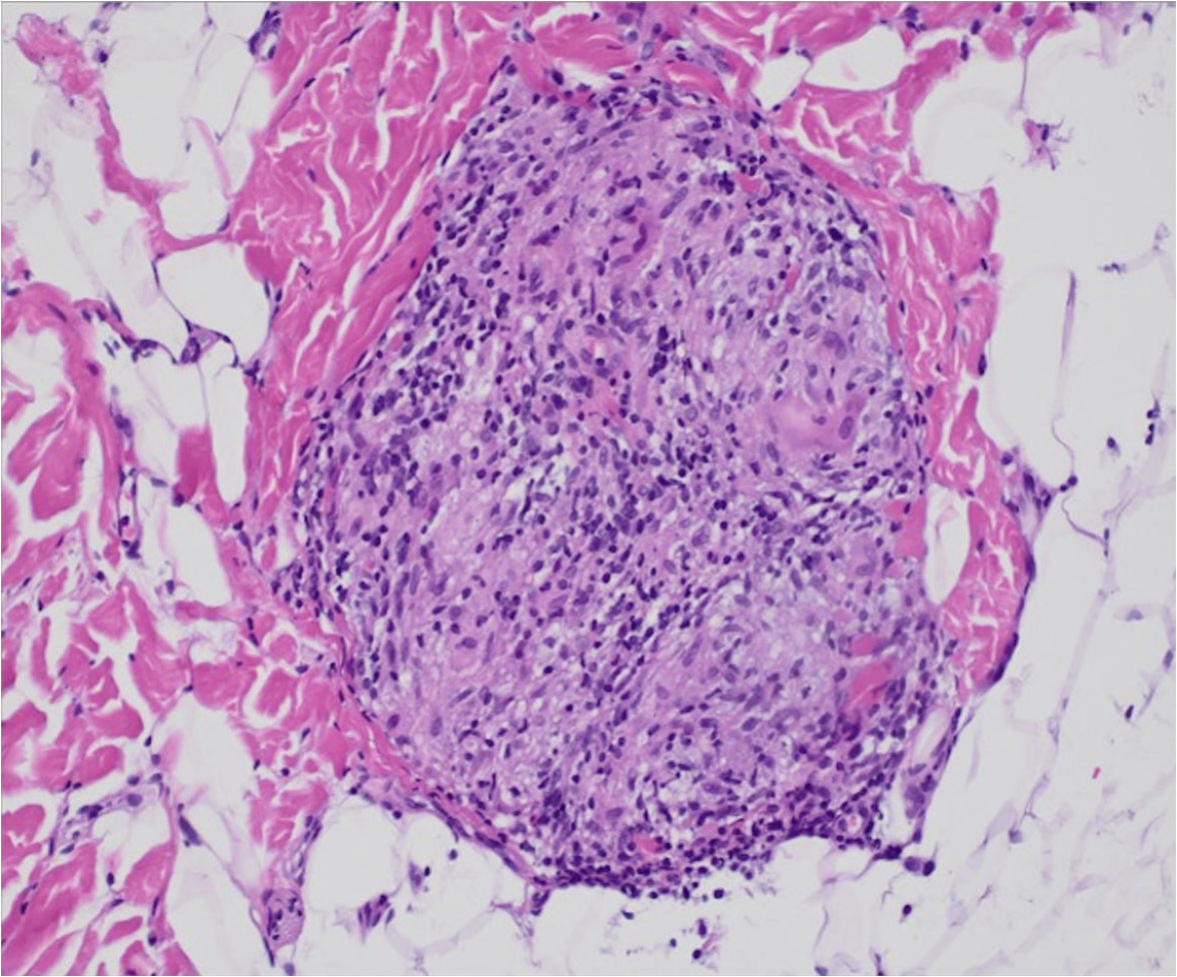
Representative image (200×) of a hematoxylin and eosin-stained section of skin from a punch biopsy from the right shoulder. Sections revealed numerous rounded aggregates of epithelioid histiocytes and multinucleated giant cells without a significant lymphocytic component (“naked” granulomas).

A 13-month-old Hispanic boy presented with a sudden-onset papular eruption affecting the face, trunk, and extremities that progressively worsened over 2 months (**Figure 1A** initial presentation; **Figure 1B** shows cutaneous presentation at 2 months after initial presentation). Prior medical history was only significant for heterochromia of the right eye (also present in the mother). The mother had no known history of NOD2 mutation or sarcoidosis. The patient was experiencing frequent falls, conjunctival injection, and apparent joint and eye pain. He had no history of difficulty breathing, cough, wheezing, blurry vision, international travel, or frequent infections. On physical exam, he had innumerable erythematous to skin-colored papules coalescing into plaques over the arms, back, legs, and face. Laboratory evaluation revealed an immunoglobulin panel with normal IgG at 799 mg/dL (normal range 480–965 mg/dL), low IgA at <50 mg/dL (normal range 52–141 mg/dL), and high IgM levels at 203 mg/dL (normal range 34–120 mg/dL). T-spot was negative. The angiotensin converting enzyme (ACE) level was elevated to 145 μg/L (ref: 18–95 μg/L). Biopsy of skin from the right shoulder was performed and revealed rounded aggregates of epithelioid histocytes and multinucleated giant cells without a significant lymphocytic component (“naked” granulomas) (**Figure 2**). Histiocytes were negative for S100 and CD1a, arguing against a Langerhans cell histiocytosis. Histocytes were diffusely positive for CD163. Special stains to rule out infection were performed, including Gram stain, acid fast stain (Ziehl–Neelsen), and Fite stain, which were negative for bacteria and mycobacteria. The patient was referred to an ophthalmologist who directly visualized subconjunctival nodules (patient could not tolerate slit lamp examination). Given the arthritis, subconjunctival nodules, and sarcoidal granulomas on skin biopsy, the patient underwent genetic testing. A mutation in codon 1000 C > T (protein R334W) in the NOD2 gene was discovered. Testing for other immunodeficiency-related genes (more than 95 other genes) showed negative results. The patient was started on prednisolone 2 mg/kg/day for 8 weeks. The patient experienced reduction in skin papules to erythematous to light-brown macules and reduction in joint swelling (assessed via inspection and palpation as well as functional improvement in mobility) and reduction in conjunctival injection and appearance of subconjunctival nodules (assessed via visual inspection) after 8 weeks of the prednisolone therapy. Two months after stopping this initial round of steroids, the patient redeveloped erythematous papules coalescing into plaques, bogginess within joints with swelling and impaired range of motion, and conjunctival injection consistent with uveitis. At this time, a second trial of repeated prednisolone 2 mg/kg/day for another 8 weeks resulting again in improvement in papular skin eruption (assessed via visual inspection), bogginess to joints and impaired range of motion (assessed via inspection and palpation by dermatology and rheumatology), and uveitis (assessed via visual inspection by dermatology and ophthalmology). Upon finishing the second round of 8 weeks of 2 mg/kg/day of prednisolone, a tapering process was performed over the next 2 weeks (10 mg daily for 7 days, then 5 mg daily for 7 days), at which time he was started on methotrexate 7.5 mg subcutaneously weekly with 1 mg folic acid orally daily. After 8 weeks, the patient experienced an improvement in his papular skin eruption to faint brown macules but continued to have significant joint swelling and arthritis (assessed on joint palpation of knees and ankles and reports by parents of persistence in difficulty with mobility) and extreme pruritus (per reports from parents) and persistent mild conjunctival erythema (assessed via visual inspection). Although pruritus is not a specifically recognized symptom of Blau syndrome, the authors assessed that the pruritus was likely a secondary symptom in response to the inflammation present within the skin, triggering the sensation of itch as is commonly experienced in a variety of dermatitides, including atopic dermatitis, psoriasis, and autoimmune connective tissue disease patients, for example. In the authors’ experience, hydroxychloroquine has helped suppress a variety of cutaneous inflammatory processes correlating with a reduction in pruritus. In consultation with rheumatology, the patient was started on hydroxychloroquine 75 mg orally daily in combination with 7.5 mg methotrexate subcutaneously weekly in combination for 8 weeks, leading to complete resolution of dermatitis (assessed via visual inspection) (**Figure 1C**), pruritus (per parents' reports), arthritis (assessed via visual inspection and palpation by dermatology and rheumatology) and uveitis (assessed via visual inspection by dermatology and ophthalmology). In discussion with the ophthalmologist, hydroxychloroquine was discontinued after the 8 weeks due to concern for risk of macular toxicity or other ocular complications and with the hope that methotrexate alone would continue to control the disease relapse from that point forward. As of the time of writing of this report, the patient continues on methotrexate 7.5 mg subcutaneously weekly with 1 mg folic acid orally daily, with appropriate follow-up with the dermatologist, rheumatologist, and ophthalmologist. The timeline of his disease status and treatment course is included in **Table 1**.

**Table 1:** Timeline of the disease status and resulting/corresponding therapy in our case.

Age	Disease status	Therapy
15 months	NOD2 mutation discovered. Severe papular dermatitis, pruritus, arthritis, uveitis	Started on oral prednisolone 2 mg/kg/day for 8 weeks
17 months	Resolved dermatitis, pruritus, arthritis, and uveitis	Finished first course of oral steroids
19 months	Recurrence of dermatitis, pruritus, arthritis, and uveitis	Re-initiation of second course of oral prednisolone 2 mg/kg/day for 8 weeks
21 months	Resolved dermatitis, pruritus, arthritis, and uveitis	Finished second course of corticosteroids
22 months	Recurrence of dermatitis, pruritus, arthritis, and uveitis	Weaned off steroids Started on 7.5 mg methotrexate subcutaneously weekly + 1 mg folic acid orally daily
24 months	Improved dermatitis, but persistent arthritis, pruritus, and uveitis	Started 75 mg daily hydroxychloroquine
26 months	Resolved dermatitis, pruritus, arthritis, and uveitis	Hydroxychloroquine discontinued due to desire to minimize long-term macular toxicity/risk for retinopathy Continued on methotrexate 7.5 mg subcutaneously weekly
30 months	Remains free of dermatitis, pruritus, arthritis, and uveitis	Continuing 7.5 mg methotrexate subcutaneously weekly + 1 mg folic acid orally daily

## Diagnostic assessment, therapeutic intervention, follow-up, and outcomes (CARE guidelines) and discussion

The diagnosis of Blau syndrome is primarily a clinical diagnosis, with supportive histomorphology, laboratory, genetic, and imaging studies, characterized by the clinical triad of dermatitis, arthritis, and uveitis (7, 9). Often mistaken for atopic dermatitis, the rash is characterized by scaly, erythematous, to skin-colored fine papules which coalesce into plaques. They can often appear as “dirty” tan-colored and scaly papules with disease progression (3). Other rarer skin manifestations include erythema nodosum, leukocytoclastic vasculitis, and livedoid-type rashes (2, 5, 6, 10, 11).

Arthritis presentation occurs in greater than 90% of cases, typically polyarticular involving both small and large joints and present with “boggy” arthritis leading to exuberant tenosynovitis (2). Range of motion is typically preserved until late disease when joint contractures can develop. Uveitis is a rarer manifestation of disease but is associated with significant morbidity in patients with Blau Sundrome (BS) (12). It occurs in approximately 60%–80% of patients and typically appears around 5 years of age. Bilateral panuveitis is the most common presentation, although variations in presentation can also occur (12). Prolonged, untreated disease leads to ongoing active inflammation and subsequent visual impairment. Common complications associated with uveitis include band keratopathy, posterior synechiae, increased intraocular pressure, and cataracts (2, 12). Nummular corneal subepithelial deposits and multifocal chorioretinal lesions have also been reported (2, 12).

Uncommon disease manifestations can occur in up to half of patients, including fever and hypertension due to non-vascular renal disease and large vessel vasculitis (2, 7, 9). Hypercalcemia has been reported in association with nephrocalcinosis and osteosclerosis. Blau syndrome-associated interstitial lung disease has been reported, manifested by cervical and mediastinal lymphadenopathy with small areas of ground glass opacities (2). Laboratory and imaging studies are not diagnostic, but they can be supportive. Serum angiotensin converting enzyme (ACE) is produced by epithelioid cells and macrophages within granulomas, and ACE elevation has been reported but can be normal (2).

The most common initial induction therapy is high-dose corticosteroids. However, due to poor disease control and an undesirable long-term side effect profile with only corticosteroids, use of other immunosuppressive or biologic agents with corticosteroids has been shown to control disease progression; promising results have been reported with methotrexate (13, 14), hydroxychloroquine (15–18), TNF-α (4, 5, 11), and IL-1 inhibitors (19). Overall, improved therapeutic outcomes are seen with early diagnosis and intervention.

We herein report a case in which our patient tolerated 8 weeks of methotrexate and hydroxychloroquine in combination, which led to the clearance of dermatitis and pruritus, arthritis, and uveitis prior to a return to methotrexate therapy only. To our knowledge, the use of methotrexate in combination with hydroxychloroquine for the treatment of Blau syndrome has not been shown in the literature. Hydroxychloroquine therapy has been shown to improve sarcoidosis-associated uveitis and may improve “neurosarcoidosis,” potentially being relevant to the persistent pruritus in our case (15, 18). Two cases reported by Milman described the use of hydroxychloroquine alone for 6 months, which was not associated with improvement (4). Hydroxychloroquine’s mechanism of action is not fully known, but it is thought to act as a weak base that is suggested to accumulate within acidic vesicles, such as lysosomal compartments leading to the inhibition of autolysosome activation and MHC class II antigen presentation; it also interferes with toll-like receptor signaling and activation (15, 17, 18). It has also been shown to inhibit the production of IL-1, IL-6, TNF-α, and INF- γ (15, 17, 18). Methotrexate is well known to suppress the immune system via inhibition of dihydrofolate reductase, leading to a broad improvement of many proinflammatory conditions, with the risk of end-organ damage and risk of a variety of infections. The tolerability of methotrexate in combination with hydroxychloroquine has not been reported in Blau syndrome; our case suggests it may be tolerated over the course of 8 weeks to improve refractory pruritus, arthritis, and uveitis. One potential and conceptual benefit of hydroxychloroquine is that it is not associated with as much of an increased risk of infectious complications due to acting as an immunomodulatory drug rather than immunosuppressive (15, 17, 18). Side effects are gastrointestinal in nature including nausea, vomiting, diarrhea, and abdominal discomfort. The most severe complication with antimalarial therapies such as hydroxychloroquine is the development of retinopathy or macular toxicity due to disruption of lysosomal degradation in the retinal pigment epithelium. It is important to note that this complication has not been confirmed with sarcoidal uveitis (17, 18). Rather, hydroxychloroquine has been shown to be a successful therapeutic in the treatment of sarcoidosis-associated uveitis (18).

Combination methotrexate-hydroxychloroquine therapy has been used in other inflammatory diseases, such as rheumatoid arthritis (16). Given the concern regarding ocular toxicity, the maximum dosages of chloroquine and hydroxychloroquine should not exceed 3.5 and 6.5 mg/kg/day, respectively. Methotrexate is given in weekly doses of 10–30 mg, with the caveat that hematological, gastrointestinal, pulmonary, and hepatic toxicities are possible. In pediatric patients, such as our case, 7.5 mg methotrexate weekly appears to be sufficient to control disease after remission was obtained with the methotrexate–hydroxychloroquine combination.

Due to the rarity of Blau syndrome and the overall limited data set in the literature, additional case reports and larger clinical studies are still needed to identify the most effective therapeutic strategies. Our case demonstrates the tolerability and effectiveness of the combination of 7.5 mg methotrexate subcutaneously weekly, 1 mg folic acid orally daily, and 75 mg hydroxychloroquine orally daily in control of dermatitis, pruritus, arthritis, and uveitis, all post-steroid treatment, with no significant reported side effects. As in our case, there may be a role for temporary combination of methotrexate and hydroxychloroquine therapy for 8 weeks, prior to reduction of therapy to methotrexate alone, depending on the effects on dermatitis, pruritus, arthritis, and uveitis, along with multidisciplinary assessment from dermatology, rheumatology and ophthalmology.

## Data availability statement

The original contributions presented in the study are included in the article/supplementary material. Further inquiries can be directed to the corresponding author.

## Ethics statement

Written informed consent was obtained from the minor(s)’ legal guardian/next of kin for the publication of any potentially identifiable images or data included in this article.

## Author contributions

MJ: Writing – original draft, Writing – review & editing. KH: Visualization, Writing – review & editing. JM: Conceptualization, Supervision, Visualization, Writing – review & editing.
